# Editorial: Targeted drug discovery in ectopic calcification: mechanism, prospect, and clinical application

**DOI:** 10.3389/fphar.2023.1159334

**Published:** 2023-05-02

**Authors:** Kang Xu, Charareh Pourzand, Jing Xie, Anna Malashicheva

**Affiliations:** ^1^ College of Pharmacy, Hubei University of Chinese Medicine, Wuhan, China; ^2^ Department of Life Sciences, University of Bath, Bath, United Kingdom; ^3^ West China Hospital of Stomatology, Sichuan University, Chengdu, China; ^4^ Institute of Cytology, Saint-Petersburg, Russia

**Keywords:** ectopic calcification, calcium deposition, therapeutic drugs, soft tissues, targeting

Ectopic calcification is a pathologic deposition of salts or bone growth in soft tissues. It occurs around large joints, such as the hip and elbow, and in various soft tissues, including blood vessels (Persy and D'Haese, 2009), heart valves (Xu et al., 2020), lungs (Bendayan et al., 2000), and kidneys (Priante et al., 2019) Although oxidative stress and inflammation have been identified as the primary determinants of soft tissue calcification, it is initiated and regulated by a series of still poorly understood molecular signaling pathways. Consequently, it is necessary to elucidate the molecular mechanism underlying ectopic calcification and to identify potential drugs to prevent its occurrence and progression.

In this Research Topic, several diseases associated with ectopic calcification, such as aortic valve calcification, pseudoxanthoma elasticum, vascular calcification, keloid fibrosis, renal fibrosis, liver cirrhosis, and Duchenne muscular dystrophy, are discussed. In light of the aforementioned diseases associated with ectopic calcification, their pathogenesis and the search for targeted therapeutic drugs were investigated in depth.

Among these, cardiovascular calcification disease research is a hot Research Topic. Bogdanova et al. provided a summary of the evaluation of aortic valve calcification in *ex vivo*, *in vitro*, and *in vivo* settings. They emphasized the translational studies of calcific aortic valve stenosis with a particular emphasis on human primary cell cultures, which are widely used and appropriate for screening anti-calcification drugs. Murtazalieva et al. evaluated the prognostic significance of valvular inflammation and calcification as measured by ^18^F-fluorodeoxyglucose (^18^F-FDG) and ^18^F-fluoride (^18^F-NaF) PET/CT in patients with tricuspid (TAV) and bicuspid aortic valves (BAV). In the case of TAV, they viewed 18F-NaF PET/CT as a more accurate and valuable predictor of the hemodynamic progression of calcific AS. Niu et al. demonstrated that BMP type I receptor A (BMPR1A) participates in osteogenic differentiation and is a potential molecular target for preventing vascular calcification. Moreover, Pan et al. reviewed recent research progress on the relationship between various types of sirtuins and vascular calcification and concluded that once a deeper understanding of the sirtuin family is established, researchers will be able to identify the most effective therapeutic targets and develop clinically applicable drugs for the prevention and treatment of vascular calcification.

Exploring and analyzing the pathogenesis of calcified cardiovascular disease has led to the discovery of numerous useful therapeutic drugs. Statins, immunosuppressants, and anticoagulants were identified by Wen et al. as potential candidates for preventing bioprosthetic heart valve (BHV) calcification. Statins such as rosuvastatin and atorvastatin may significantly reduce calcification of BHV. In addition, anti-thymocyte globulin (ATG), a polyclonal IgG preparation used for immunosuppression induction, uncovered a potential therapeutic strategy for preventing BHV calcification. In addition, the European and American Association Guidelines recommend oral anticoagulation for the first 3 months following surgical implantation of BHV. Huang et al. discovered that andrographolide, a natural terpenoid extracted from the traditional Chinese medicinal plant *Andrographis paniculate*, reduces aortic valve enlargement by inhibiting cell proliferation and osteogenic differentiation through the MAPK-ERK signaling pathway. FLT3 was identified by Wang et al. as a target protein that contributed to calcified aortic valve disease. They demonstrated that Atractylenolide-1, a natural active compound extracted from *Atractylodes macrocephala*, inhibited the phosphorylation of FLT3, thereby blocking the activation of the PI3K/AKT pathway, reducing the production of Hypoxia-inducible factor (HIF)1-α, and subsequently inhibiting the osteogenic differentiation of valve interstitial cells (VICs). In addition, Zhang et al. confirmed that dihydromyricetin (DHM), the most bioactive component of *Ampelopsis grossedentata*, significantly inhibited the osteogenic differentiation of human VICs by inhibiting c-KIT phosphorylation and suppressing IL-6 expression. DHM is an effective pharmacological treatment for preventing the progression of calcified aortic valve disease.

Ectopic myofiber calcification is a pathological characteristic of muscle damage in Duchenne muscular dystrophy (DMD). According to Rumney et al., P2X7 gene overexpression serves as a protective mechanism against dystrophic mineralization. In cultures of dystrophic cells, the use of the P2X7 agonist BzATP decreased the calcifying effects of high phosphate that promoted mineral deposition. Additionally, persistent calcium deposits in the kidneys result in inflammation and cell necrosis, which are associated with severe kidney diseases. Feng et al. developed a predictive drug target method for the diagnosis of renal fibrosis and identified the EGR1 and PLA2G4A genes as the targets for this calcific-associated disease. Chi et al. investigated the diagnostic key targets of tanshinone IIA, which is associated with serum calcium levels, in liver cirrhosis. After bioinformatic analysis, AKR1C3 and TPX2 were identified as the molecular targets involved in the development of liver cirrhosis. Biomechanical regulatory factors also contributed to ectopic calcification, which is quite interesting. Feng et al. provided a summary of the mechano-transduction pathways, such as the TGF-β/Smad signaling pathway, the integrin signaling pathway, and the YAP/TAZ signaling pathway, that are involved in the formation of keloids, which results in excessive collagen disorders and calcinosis. By targeting biomechanical regulatory factors, these findings may facilitate the development of pharmacological interventions for treating ectopic calcification.

In the process of investigating medications for the treatment of ectopic calcification, we will face numerous obstacles. Anti-calcification medications may inhibit bone regeneration or cause osteoporosis. In the recovery period following a bone fracture, elderly patients who must take drugs for the treatment of ectopic calcification diseases face this dilemma. In the subsequent phase of research, it will be crucial to develop drugs that target ectopic calcification without affecting bone mineralization. In addition, for specific cardiac calcifications, such as aortic valve calcification, it is difficult to construct a stable organoid for basic research, such as drug screening or calcification mechanism development, because the cells involved in calcification in valve tissue are difficult to obtain. This lack of a reliable *in vitro* calcification model has severely impeded the progress of anti-ectopic calcification research.

The pathogenesis of ectopic calcification diseases is relatively complicated, involving the activation of multiple molecular signaling pathways and functional genes, the disorder of cellular metabolism, fibrosis of the extracellular matrix, and calcium deposition (Collett and Canfield, 2005; Gonzalo and Villa-Bellosta, 2019). Exploring the underlying mechanisms of ectopic calcification, followed by screening and identifying therapeutic drugs based on associated signal pathways and molecular targets, is an applicable research model for identifying potential drugs for preventing ectopic calcification.
